# Health-related quality of life in pediatric liver transplanted patients compared with a chronic liver disease group

**DOI:** 10.1186/1824-7288-39-55

**Published:** 2013-09-11

**Authors:** Antonella Gritti, Simone Pisano, Tiziana Salvati, Nicolina Di Cosmo, Raffaele Iorio, Pietro Vajro

**Affiliations:** 1 Faculty of Education Science, Suor Orsola Benincasa University, Naples, Italy; 2 Department of Mental and Physical Health and Preventive Medicine, Child and Adolescent Psychiatry Division, Second University of Naples, Naples, Italy; 3 Department of Pediatrics, University of Naples Federico II, Naples, Italy; 4 Department of Medicine and Surgery, University of Salerno, Salerno, Italy

**Keywords:** Paediatric liver transplantation, Quality of life, Chronic disease

## Abstract

**Background:**

Achieving a good health-related quality of life (HRQoL) is currently one of the main aims in long term survival of liver transplanted children (PLT). Purpose of our study is to compare HRQoL of PLT patients (N = 33, mean age 12.8 y) vs. sex and age matched patients with compensated and clinically stable chronic liver disease (CLD) (N = 25, mean age 11.9 y).

**Methods:**

HRQoL was measured from both patient and parental perspectives using the CHQ-CF87 and CHQ-PF50 questionnaires, respectively.

**Results:**

General Health Perception scores of PLT subjects resulted significantly lower than those of CLD both at self- and parental report (p < 0.05 and p < 0.01, respectively). No other significant differences in other HRQoL domains were found between groups.

**Conclusion:**

Our results suggest that the two populations are quite similar regarding HRQoL, but both parents and children of PLT group perceive a worse general health. Further studies are needed to confirm these results.

## Background

Liver transplantation (LT) has become the treatment of choice for several pediatric liver diseases that progress to end-stage liver failure. Currently patients long-term survival rates are reported to be between 85% and 90% [1]. The possibility of prolonging recipient life expectancy has triggered several studies focusing not only on physical problems but also on psychological and psychosocial outcomes. At the moment, the evaluation of Health Related Quality of Life (HRQoL) after pediatric liver transplantation (PLT) is one of the main focuses of several researches. It is to note that patients who receive transplantation face a set of combined conditions: a threatening liver disease, the transplant surgery, and the post-transplant assistance. As a consequence, children and families are exposed to a long-standing stressful process that may influence their Quality of Life (QoL). As known, HRQoL is a multidimensional construct comprising several domains. Appropriate assessment of HRQoL includes both patient and parents perspectives in five domains: physical health, mental health, social functioning, role functioning and general health perception.

Studies on HRQoL after PLT are essential and have a large impact on assistance, since they can provide information also about outcomes and factors related to non-adherence [2].

Several studies on HRQoL after PLT have been published. Most of them compared samples of PLT to healthy children rather than to chronically ill matched controls. It has been suggested that PLT recipients report a poorer HRQoL in comparison with healthy population [3-8]. In particular, physical and psychosocial domains and school functioning are worse than those of the normative samples. These results are not surprising because children who received liver transplantation and their families experienced much more stressful events than healthy peers. Taylor reviewed literature on HRQoL of children and adolescents after liver transplantation and reported five studies that included a comparison group of chronically ill population. On the basis of these studies, authors concluded that: *“HRQL after liver transplantation tended to be equal to or better than in children and adolescents with other chronic illness”*[5]. However, other studies have explored this issue [2,7,9-11] with at odd conclusions. For example, Fredericks [2], by comparing PLT children with a control group of children affected by cancer or diabetes, demonstrated that PLT group achieved lower school functioning, total physical and psychosocial scores than chronic group. Limbers [11] conducted a study on a large cohort of 873 PLT subjects and compared the sample with a control group including several chronic diseases (Juvenile Rheumatoid Arthritis, Diabetes, Cancer, Cardiac disease and Renal transplant). Final data showed that PLT patients had an impaired HRQoL similar to chronic diseases ones with even lower school functioning. Moreover, post-transplant HRQOL evaluated *vs*. pre-transplant group of patients in a waiting list improved dramatically after LT [12].

Finally, Duffy [10] studying HRQoL of young adults 20 years after transplantation showed that their HRQoL was significantly better than in patients with chronic liver disease (CLD). To our knowledge, this study is the only one of those mentioned above, which includes a comparison group affected by a CLD. In a previous investigation [13], our group explored psychological and psychopathological consequences of PLT pediatric patients compared with CLD controls. By using CBCL-PRF (Children Behaviour Check List-Parent Form) we demonstrated an higher risk for psychological problems reported by parents of PLT group.

On this basis, here we hypothesize that PLT children could have poorer HRQoL than CLD patients. We assume that comparison between PLT patients and CLD samples may be useful to understand effects of transplant process on HRQoL.

According with above hypothesis, the aim of the present research was to assess HRQoL in a sample of PLT patients compared with a CLD group.

## Methods

Candidates to enrollment were patients, aged 5–18 years, who had undergone liver transplantation for end-stage liver failure and regularly followed up at the Department of Pediatrics of the University of Naples ‘Federico II’ ^a^.

Exclusion criteria were: acute disease needing intensive care, intellectual disability (IQ score below 70, assessed by a trained clinical psychologist through Wechsler Intelligence Scale for Children, WISC, or Wechsler Adult Intelligence Scale, WAIS, according to age).

Participants were recruited among PLT patients followed-up by pediatricians during the period of October 2007 – January 2009.

36 subjects were eligible for the study. 2 of them (with perinatal asphyxia) were excluded because they did not meet inclusion criteria (IQ below 70). In one case family refused to participate. Globally, 91% of total candidates were included in the study.

As shown in Table 1, the final sample was composed of 33 PLT subjects (F:14, M:19), aged between 6.2-18 years, (mean age 12.82 yrs, SD 3.68). Mean age at PLT was 3.5 yrs SD 3.7. According to manual, 3 of 33 subjects younger than 10 could not answer the questionnaire. PLT group finally comprised 30 children, while parents group was composed by 33 subjects.

**Table 1:** Demographics of PLT and CLD patients

	**PLT group**	**CLD group**	**P**
**Number of subjects**	33	25	-
**Gender, M(%)-F(%)**	19 (58%) – 14 (41%)	13 (51%) -12 (49%)	0.79
**Age** **(range, mean, sd) (years)**	6.2-18.0;12.8; sd ± 3.6	6.6-18.0; 11.9 yrs; sd ± 3.0	0.316
**Age at transplantation (range, mean, sd) (years)**	0.2-12.5; 3.5; sd ± 3.7	-	
**Avg time elapsed from PLT (mean, sd) (years)**	12.1; sd ± 4.3	-	
**Average rate hospitalization (admissions/year) (mean, sd)**	7.9; sd ± 3.7	Mean 2.9; sd ± 1.06	**0.0001**
**Race (language)**	Caucasian (Italian)	Caucasian (Italian)	
**Indications for liver transplant**	Biliary atresia (n = 29); Metabolic liver disease (n = 2); Autoimmune hepatitis (n = 1); Hepatoblastoma (n = 1)	-	
**Type of chronic liver disease**	-	Chronic viral or autoimmune hepatitis (n = 13); Wilson’s disease (n = 6); Cryptogenic chronic hepatopathy (n = 5); Others (n = 1).	

*PLT* pediatric Liver Transplant group, *CLD* chronic Liver Disease group.

CLD group was selected among chronic hepatopatic patients followed up at the same Department on the basis of age and gender matching with PLT group, and at least 1 year long clinically stable chronic liver disease (as supported by medical history, physical examination, and laboratory tests). It comprised 25 subjects (F:12, M: 13) aged between 6.6-18.0 years (mean age 11.9 years, SD 3.0) (Table 1). At the time of the study all were in good clinical compensation, without signs of portal hypertension. None of them was in LT waiting list. No patient with viral hepatitis was on drug therapy, whereas patients with Wilson disease and Autoimmune hepatitis where taking penicillamine or steroids, respectively.

According to manual, 2 of 25 subjects younger than 10 could not answer the questionnaire. Therefore, CLD group finally comprised 23 children, while parents group was composed by 25 subjects.

### Procedures

Parents were informed by the pediatric hepatologists about the aims and methods of the research and were asked if they would be willing to participate. After obtaining informed consent, children and parents were assessed separately by a child psychiatrist in a separate setting from the pediatric hepatology section. Parents and subjects of both groups involved in the study - PLT and CLD - were invited to answer their respective questionnaires. Preferably parents were asked to give agreed answers on the same questionnaire sheets; in case of high disagreement, the caregiver spending more time with the child was preferred. Procedures are in accordance with the Helsinki Declaration of 1975.

### Measures

Subjective Quality of Life was assessed through the Child Health Questionnaire (CHQ) [14], an internationally accepted general Quality of Life survey. Rigorously developed and validated with numerous publications, psychometric proprieties of the Italian CHQ version have been demonstrated, as well [15]. Both a child (CHQ-CF 87) and caregiver version (CHQ-PF 50) are available. The CHQ-PF 50 are designed for self-completion by parent/guardian of the child. They have been validated for use with children at least five years of age or older. The CHQ-CF 87 is designed for self-completion by children at least ten years of age. Both have been tested in normative populations as well as in children with a wide variety of chronic diseases. Both versions have demonstrated strong internal consistency and validity across diverse clinical groups. Areas measured include: *General Health Perception* (perception of overall health), *Physical Functioning* (physical limitations attributable to health-related problems), *Role-physical (*limitations in school-work and activities with friends), *Behavior* (aggression, delinquency, hyperactivity/impulsivity, and social withdrawal), *Mental Health* (anxiety, depression, and positive affect) *Role-emotional (*limitations in activities with friends and at school caused by emotional or behavioral problems), *Parent Impact Emotional* (distress experienced because of child’s condition), *Parent Impact Time* (limitations in parent’s personal time because of child’s status), *Family Cohesion* (how well family members get along with one another), *Bodily Pain* (intensity and frequency of general pain and discomfort), *Self-Esteem (*satisfaction with school and athletic ability, looks or appearance, ability to get along, and feelings about life overall); *Family activities (*frequency of disruption of usual family activities). Further measures are the two summary scales Physical Score (PhS) and Psychosocial Scores (PsS). Scores are standardized to 0–100, with higher scores indicating better well-being.

According to User’s Manual, CHQ response options vary. For example most scales ask about the past 4 weeks whereas for the “Global Health” and “Family Cohesion“ items asking about health and family relationship “in general” no recall period is used.

### Statistical analysis

CHQ scoring was performed according to the manual of CHQ-CF87 and CHQ-PF50.

Fisher’s exact test and two paired t-test were used for demographic characteristics between groups, respectively for categorical and continuous variables.

Data of PLT sample were compared with those obtained from gender and age matched children affected by CLD. As we do not assume *a priori* that scores of both groups were normally distributed, a non-parametric analysis (Mann–Whitney test) was used for the comparison. P value < 0.05 was considered statistically significant.

Effect sizes for differences in means are designated as small (0.20), medium (0.50), and large (0.80) in magnitude.

Study of correlation between results at CHQ-CF87 and age, age at transplant, time elapsed from transplant was conducted with the Spearman’s rank correlation test.

## Results

Refusal or ineligibility rate was negligibly low (3 PLT patients).

Results of CHQ-CF87 are summarized in Table 2. Scoring CHQ-CF87 the following results were obtained: PLT subjects reported significantly lower scores than CLD in General Health Perception subscale (p < 0,05). Results in other subscales didn’t reach statistically significant differences.

**Table 2:** Child Health Questionnaires (CHQ-CF87) scores in PLT group versus CLD group

	**PLT (n = 30)**	**CLD (n = 23)**	**PLT (n = 30)**	**CLD (n = 23 )**	**P**	**Effect Size (d)**
	**Mean ± SD**		**Median (Range)**			
**Physical Function (PF)**	86.2 ± 14.2	87.2 ± 20.4	92 (44–100)	92 (0–100)	0.57	−0.05
**Role Emotional (RE)**	85.1 ± 17.2	90.4 ± 20.8	88 (33–100)	100 (0–100)	0.07	−0.27
**Role Behavior (RB)**	85.1 ± 18.6	87.5 ± 17.2	88 (33–100)	88 (33–100)	0.65	−0.13
**Role Physical (RP)**	86.3 ± 19.0	89 ±16.4	100 (33–100)	100 (33–100)	0.72	−0.15
**Bodily Pain (BP)**	72 ± 26.5	70.6 ± 24.1	80 (0–100)	80 (20–100)	0.78	0.05
**Behavior (BE)**	76.5 ± 15.7	72.5 ± 17.5	78 (42–100)	75 (42–100)	0.49	0.24
**Mental Health (ME)**	72.6 ± 16.0	75.6 ± 15.6	72 (32–100)	78 (29–92)	0.24	−0.18
**Self Esteem (SE)**	82 ± 11.1	81.8 ± 10.6	85 (55–98)	82 (54–99)	0.78	0.01
**General Health Perception (GH)**	**32.1 ± 12.5**	**44.6 ± 22.4**	**33 (6–56)**	**42 (13–93)**	0.03*	**−0.68**
**Family Activities (FA)**	80.9 ± 22	77.3 ± 18.8	87 (20–100)	79 (37–100)	0.23	0.17
**Family Cohesion (FC)**	87 ± 18.2	76 ± 28.7	100 (30–100)	85 (0–100)	0.17	0.45
**Physical (PhS)**	44.6 ± 6.4	45.8 ± 8.1	43 (34–56)	46 (18–58)	0.2	−0.16

*PLT* pediatric Liver Transplant group, *CLD* chronic Liver Disease group.

*p < 0,05.

Similarly, CHQ-PF50 filled by PLT parents (Table 3) scored significantly lower than those of CLD parents in General Health Perception (p < 0,01) subscale*.* Results in other subscales didn’t reach levels of significance.

**Table 3:** Child Health Questionnaires (CHQ-PF50) scores in PLT group versus CLD group

	**PLT (n = 33)**	**CLD (n = 25)**	**PLT (n = 33)**	**CLD (n = 25)**	**P**	**Effect Size (d)**
	**Mean ± SD**		**Median (Range)**			
**Role Emotional Behavior (REB)**	78.4 ± 29.3	76.5 ± 29.0	88 (0–100)	88 (0–100)	0.66	0.06
**Role Physical (RP)**	90.3 ± 20.1	77.8 ± 31.5	100 (16–100)	100 (0–100)	0.14	0.47
**Bodily Pain (BP)**	82.4 ± 19.8	74.4 ± 26.0	80 (20–100)	80 (40–100)	0.2	0.34
**Behavior (BE)**	70.7 ± 15.3	75 ± 15.0	70 (25–100)	70 (45–95)	0.3	−0.28
**Mental Health (ME)**	65.2 ± 18.8	70 ± 17.5	70 (14–90)	70 (35–100)	0.7	−0.26
**Self Esteem (SE)**	69 ± 18.5	71.7 ± 21.2	70 (25–100)	79 (20–95)	0.3	−0.12
**General Health Perception (GH)**	**44.7 ± 16.5**	**56.6 ± 17.0**	**45 (20–80)**	**60 (0–90)**	0.00*	**−0.71**
**Parental impact- emotional (PE)**	56.7 ± 24.5	64.7 ± 21.7	58 (8–100)	58 (25–100)	0.2	−0.34
**Parental impact-time (PT)**	75.4 ± 28.4	84.9 ± 19.3	77 (0–100)	88 (44–100)	0.33	−0.38
**Family Activities (FA)**	86.6 ± 13.5	82.8 ± 17.0	91 (54–100)	91 (41–100)	0.37	0.24
**Family Cohesion (FC)**	71.0 ± 25.4	70.2 ± 26.0	60 (30–100)	60 (30–100)	0.82	0.02
**Physical (Phs)**	46.8 ± 11.3	45.9 ± 10.1	50 (16–63)	47 (11–57)	0.2	0.08
**Psychosocial (PsS)**	43.1 ± 8.9	44.6 ± 11.5	43 (25–61)	48 (10–61)	0.3	−0.14

*PLT* pediatric Liver Transplant group, *CLD* chronic Liver Disease group.

*p < 0,01.

Correlations between results on CHQ-CH87 and some demographic variables (Table 4) showed positive mild or moderate correlations between age of subjects and Physical Function, Role Physical, Bodily Pain and Physical subscales. Positive mild or moderate correlations were observed between time elapsed from PLT and Physical function, Role Emotional, Role Behavioral, Role Physical, Behavior, Physical subscales. A negative moderate correlation was observed between age of subjects and Self Esteem. Negative mild correlations were observed between age at PLT and Role Emotional and Psychosocial subscales. Negative mild correlations were observed between time elapsed from PLT and Self Esteem and General Health Perception subscales.

**Table 4:** **Correlations between scores and demographic variables in PLT group (Spearman’s Rank Correlation, *ρ*)**

	**Age**	**Age at PLT**	**Time elapsed from PLT**
**Physical Function (PF)**	+0.50**	−0.03	+0.56**
**Role Emotional (RE)**	0.09	−0.28*	+0.33*
**Role Behavior (RB)**	0.18	−0.09	+0.40*
**Role Physical (RP)**	+0.44**	0.11	+0.47**
**Bodily Pain (BP)**	+0.21*	−0.06	0.16
**Behavior (BE)**	0.11	−0.16	+0.31*
**Mental Health (ME)**	−0.08	−0.3	0.12
**Self Esteem (SE)**	−0.44**	−0.01	−0.24*
**General Health Perception (GH)**	−0.19	−0.09	−0.24*
**Family Activities (FA)**	0.10	0.008	0.05
**Family Cohesion (FC)**	−0.06	0.09	0.12
**Physical (PhS)**	+0.56**	0.11	+0.49**
**Psychosocial (PsS)**	−0.2	−0.29*	0.04

*PLT* Pediatric Liver Transplant group.

*mild correlations (p < 0,05).

**moderate correlations (p < 0,01).

## Discussion

Overall results indicated that PLT children had a worse *General Health Perception (GHP)* than CLD, with comparable HRQoL in other areas. GHP effect size (Cohen’s d) resulted between moderate and high values (−0,68; -0,71). At any rate, results are strengthened by accordance between children and parents reports.

General Health perception is a subjective assessment of overall health and illness. Parents are asked to best describe their child’s past, future and current health and resistance/susceptibility to sickness.

These results are consistent with previous reported ones, summarized by Taylor [5] and highlighted by Sundaram [9]. However, Duffy et al. [10] found a better HRQoL when compared LT and CLD patients, but data came from an adult population transplanted two decades earlier. Our is the first study aimed to compare PLT and CLD from both children and parents perspective.

Data that could, at least partially, explain our findings are those regarding hospitalization rate per year. PLT patients and their families in fact tend to experience much more hospitalization time per year, possibly negatively influencing their own general health perception in respect of CLD patients. Their hospitalizations included a first year strict program of liver function monitoring with frequent need for corrections of medical and surgical complications; thereafter repeated admissions for Day Hospital and/or outpatient visits were scheduled with variable timing tailored on individual necessity.

Correlations analysis overall indicate that - differently from self-esteem- quality of life in physical, familiar and behavioral areas are positively associated with age and time elapsed from transplantation. Data regarding self-esteem in PLT however should be evaluated cautiously because this subscale did not show statistically significant differences with general population (data gathered from User Manual) and because self esteem needs to be assessed with more specific instruments. Undoubtedly, these data have to be considered still exploratory because of the relatively small sample size and the cross sectional nature of the study. All these issues should be further explored, eventually also with perspective studies. By the developmental viewpoint, we suggest that very young patients may lack the cognitive and emotional competences to promote adjustment to transplantation. On the contrary, subjects who received PLT later in the life could better cope the transplantation process.

Taking together findings from current and our group's previous study [13], we suggest that PLT patients should be considered at high risk for psychological problems, and reduced quality of life as well. As already proposed [13], a psychological support and monitoring soon after transplantation should be part of standard clinical management of PLT patients and should be addressed to prevent psychopathological risk and to support quality of life.

This study has several limitations. In addition to the small size of the sample and the single centre recruitment, the lack of healthy control group could limit our findings. However, we remind that our aim was not to compare PLT HRQoL with healthy population (whose data are provided by already existing literature), but with a chronic hepatic condition.

## Conclusion

Knowledge on psychological and psychopathological effects of PLT has much improved overtime [16,17]. More recently QoL is acquiring more and more a pivotal role to optimize patient monitoring, to the point that its assessment should be complementary to clinical and laboratory data. We believe that our study’s specific finding of a poorer HRQoL General Health Perception subscale in PLT patients respect to CLD ones could be highly relevant in order to provide them adequate psychological support, and should be taken into account during transplantation decision-making procedures [18]. In this regard a multidimensional program is recommended. Three main areas should be included: physical health (e.g. drug side effects, early detection of medical complications), mental health (e.g. self-esteem, body image, depression, anxiety), social (e.g. relationships, school).

### Endnote

^a^This study is part of a wider program on liver transplanted children, lasting since several years, based on the strict cooperation between the Department of Pediatrics of the University of Naples ‘Federico II’ and the Chair of Child Neuropsychiatry of the Second University of Naples (SUN) and also Suor Orsola Benincasa University and Chair of Pediatrics University of Salerno. The Department of Pediatrics of the University of Naples “Federico II” represents the only medical center of Campania Region involved in the diagnosis and treatment of severe liver diseases in the pediatric age, including the follow-up of children who have undergone PLT. More than 100 patients (children, adolescents and young adults) who have been transplanted in childhood as a result of a wide variety of liver diseases (cholestatic, metabolic, auto-immune, infectious) are or have been followed at these Departments. Therefore, our group is interested to identify and to prevent the multi-organ complications and, as mentioned, emotional and psychological disorders in this context.

## Competing interests

The authors declare that they have no competing interests.

## Authors’ contributions

AG, designed the study, interpreted data, drafted and revised the manuscript. SP collected data, drafted and revised the manuscript.TS analyzed data. NDC, collected clinical data. RI critically revised the manuscript. PV designed the study, interpreted data, drafted and revised the manuscript. All authors read and approved the final manuscript.
